# Highly aligned Ti_3_C_2_T_*x*_ MXene films with high anisotropic thermal conductivity

**DOI:** 10.1039/d6nr02529c

**Published:** 2026-10-19

**Authors:** Wenkang Chen, Mauro Ramos Jimenez, Kai Xu, Maria-Anna Moustaka, Katja Klinar, Diego Collado Ramos, Xiangfan Xu, Jorge Rodríguez-Moreno, Elena Navarrete-Astorga, Alexandros El Sachat, Javier Pérez Carvajal, Miguel Muñoz Rojo

**Affiliations:** a Energy2Devices, Instituto de Ciencia de Materiales de Madrid (ICMM), CSIC Madrid 28049 Spain m.m.rojo@csic.es; b Center for Phononics and Thermal Energy Science, China-EU Joint Center for Nanophononics, School of Physics Science and Engineering, Tongji University 200092 Shanghai China; c Institute of Nanoscience and Nanotechnology, National Center for Scientific Research “Demokritos” Athens Agia Paraskevi 15341 Greece; d University of Ljubljana, Faculty of Mechanical Engineering, Laboratory for Refrigeration and District Energy Askerceva 6 1000 Ljubljana Slovenia; e Departamento de Tecnología Electrónica, Escuela de Ingenierías Industriales, Universidad de Málaga 29071, Málaga Spain; f Instituto Universitario de Materiales y Nanotecnología, IMANA, Universidad de Málaga 29071, Málaga Spain; g Departamento de Física Aplicada I, Facultad de Ciencias, Universidad de Málaga 29071, Málaga Spain; h Department of Thermal and Fluid Engineering, University of Twente Enschede Netherlands

## Abstract

High-density integration in modern electronic components induces severe heat accumulation, compromising operational stability and device longevity. Traditional metallic thermal conductors are heavily restricted by their substantial mass density and poor mechanical processability in lightweight electronics, demanding anisotropic materials that can provide robust thermal insulation across the out-of-plane direction while facilitating rapid heat dissipation along the in-plane direction. Here, we fabricate scalable and highly aligned Ti_3_C_2_T_*x*_ MXene films using the minimally intensive layer delamination method followed by vacuum-assisted filtration. Employing frequency-domain thermoreflectance, we achieve a precise characterization of directional heat transport on identical samples, revealing an exceptional thermal anisotropy ratio of 85. The fabricated films exhibit a significantly suppressed room-temperature out-of-plane thermal conductivity of 0.54 ± 0.09 W m^−1^ K^−1^ and an in-plane thermal conductivity of 46 ± 6 W m^−1^ K^−1^. Temperature-dependent characterization shows that out-of-plane thermal conductivity exhibits a positive temperature coefficient up to 353 K before stabilizing into a saturation plateau near 373 K, confirming that cross-plane heat transport is fundamentally regulated by the dense interlayer physical boundaries. At the microscopic interface, surface hydroxyl groups and interfacial hydrogen-bonding networks may modify local inter-flake phonon transport channels. The in-plane thermal conductivity decreases overall with increasing temperature, reducing the anisotropy while preserving substantial directional contrast. This work provides a versatile and high-performance platform for implementing structurally oriented MXene assemblies in advanced directional thermal management.

## Abstract 2

We investigate the temperature dependent out-of-plane and in-plane thermal conductivity of highly aligned Ti_3_C_2_T_*x*_ films. The results reveal anisotropic ratios of 85, highlighting the potential of MXenes for directional thermal management.

## Graphical abstract

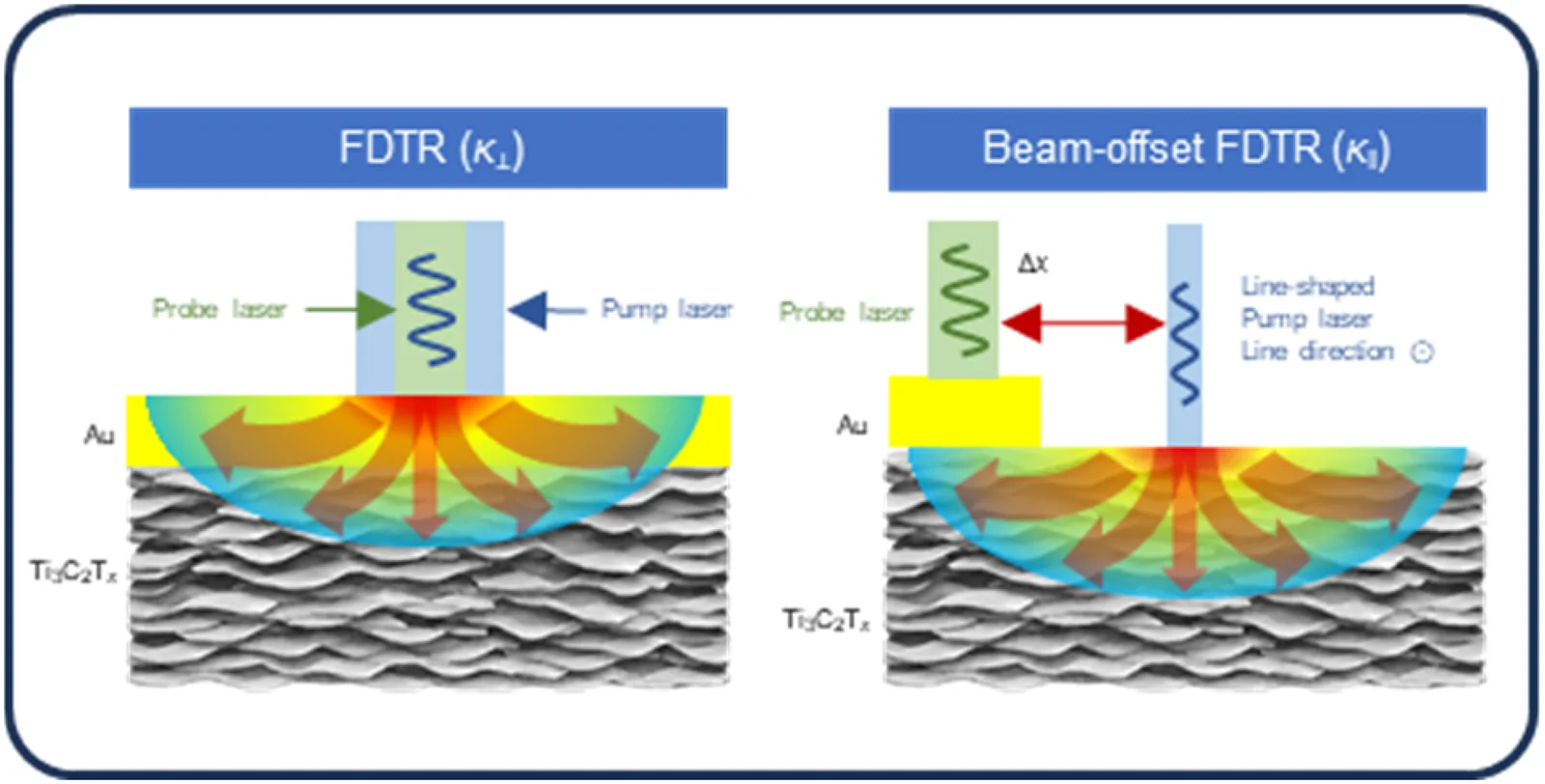

## Introduction

The rapid integration and miniaturization of modern electronic components lead to severe heat accumulation within integrated circuits. This thermal buildup reduces operational stability and shortens device lifetime, posing serious safety hazards over time.^1^ Consequently, developing reliable thermal management strategies remains universally critical to dissipate excess heat into the surrounding environment or to thermally insulate and protect sensitive electronic components. Traditional metallic conductors, such as copper and aluminum, exhibit inherently high thermal conductivity. However, their substantial mass density, high susceptibility to environmental corrosion, and poor mechanical processability heavily restrict their integration into next generation lightweight and flexible electronics.^2^ Conversely, typical lightweight polymer composites often show low intrinsic thermal conductivity, rendering them insufficient for extreme high power operating scenarios, or they degrade after exposure to high temperatures or long thermal cycles.^3^

Anisotropic thermally conductive films assembled from two-dimensional nanomaterials, like graphene,^4,5^ boron nitride^6,7^ or molybdenum disulfide,^8,9^ offer an optimal and highly adaptable solution for advanced thermal management systems. These engineered layered architectures deliver controlled and directional heat transfer capabilities. Specifically, they can facilitate rapid heat dissipation along the in-plane direction to efficiently eliminate localized hotspots. Simultaneously, they provide robust thermal insulation across the out-of-plane direction that could be used to shield heat sensitive components from parasitic thermal degradation.^10,11^ This tailored thermal anisotropy relies heavily on optimizing internal phonon transport pathways, which requires balancing an extended phonon mean free path in the basal plane with restricted transverse transport across dense interlayer boundaries.^12^

Among various two-dimensional building blocks, transition metal carbides and nitrides, broadly classified as MXenes,^13–15^ demonstrate exceptional utility for these sophisticated anisotropic thermal management applications. Theoretical calculations indicate that MXenes can present high intrinsic in-plane thermal conductivity (*κ*_∥_), where specific surface terminations critically govern their thermal response yielding *κ*_∥_ up to 108.2 W m^−1^ K^−1^.^16^ Furthermore, their unique surface characteristics facilitate direct assembly into highly ordered macroscopic films. Realizing their maximum anisotropic thermal performance in practical electronics requires precise control over macroscopic structural alignment and careful minimization of interfacial thermal resistance to prevent severe phonon scattering.^17^

In this work, we present a comprehensive investigation into the thermal transport properties of scalable and highly anisotropic Ti_3_C_2_T_*x*_ MXene films, obtaining high thermal anisotropy for heat management applications. By utilizing the minimally intensive layer delamination (MILD) method followed by vacuum assisted filtration (VAF), we successfully fabricated highly oriented macroscopic films with minimal structural defects. We employed frequency domain thermoreflectance (FDTR) in both concentric and beam-offset configurations to achieve a precise, decoupled characterization of directional heat transport on identical samples. Our results reveal a significantly suppressed out-of-plane thermal conductivity (*κ*_⊥_) of 0.54 ± 0.09 W m^−1^ K^−1^ at room temperature, which stands in stark contrast to a *κ*_∥_ of 46 ± 6 W m^−1^ K^−1^. This study demonstrates a remarkable thermal anisotropy ratio (*κ*_∥/_*κ*_⊥_) of 85, providing a foundational framework for implementing MXene assemblies in advanced directional thermal management.

## Method

### Preparation method

The fabrication of the film began with the synthesis of a colloidal stable water-based MXene solution using the MILD procedure. This route was selected as a less hazardous and aggressive approach compared with the direct HF method.^18^ For that purpose, a HF solution was formed *in situ* by adding 1.2 g of LiF and 15 mL of 9 M HCl into a PTFE bottle. The mixture was stirred magnetically for 10 minutes before 0.75 g of the Ti_3_AlC_2_ MAX phase precursor (Carbon–Ukraine Ltd) was added over a 20-minute period to avoid excessive temperature increases. The solution was stirred at 750 rpm for 28 hours at 35 °C. Following the etching process, the solution was centrifuged at 3500 rpm for 5 minutes, and the supernatant was discarded. Deionized (DI) water was added, followed by hand shaking to separate the MXene flakes from non-delaminated accordions. This washing cycle was repeated until dark green colloidal stability was reached. To enhance the synthesis yield *via* Li^+^ intercalation, the entire solution was added to 30 mg mL^−1^ of LiCl and stirred at 750 rpm for 24 hours. After a final washing cycle, a stable single-flake colloidal solution was obtained. From this stabilized solution, MXene films were fabricated using the VAF process. For each 18 mm diameter sample, the colloidal solution was redispersed with 60% water, with 45 mL of the dispersion used per film. The suspension was deposited onto a nanoporous track-etch polycarbonate (PC) membrane with a 100 nm pore size (Whatman®). A vacuum of 80 mbar was applied for 24 hours. For the conventional cross-plane FDTR measurements, a nominally 150 nm Au transducer was deposited over the MXene film surface by e-beam evaporation. A separate sapphire reference received Au in the same deposition run for transducer calibration. For the in-plane beam-offset measurements, the Au coating covered only part of the MXene surface, leaving an adjacent bare region for pump excitation, as illustrated in Fig. 1.

**Fig. 1:**
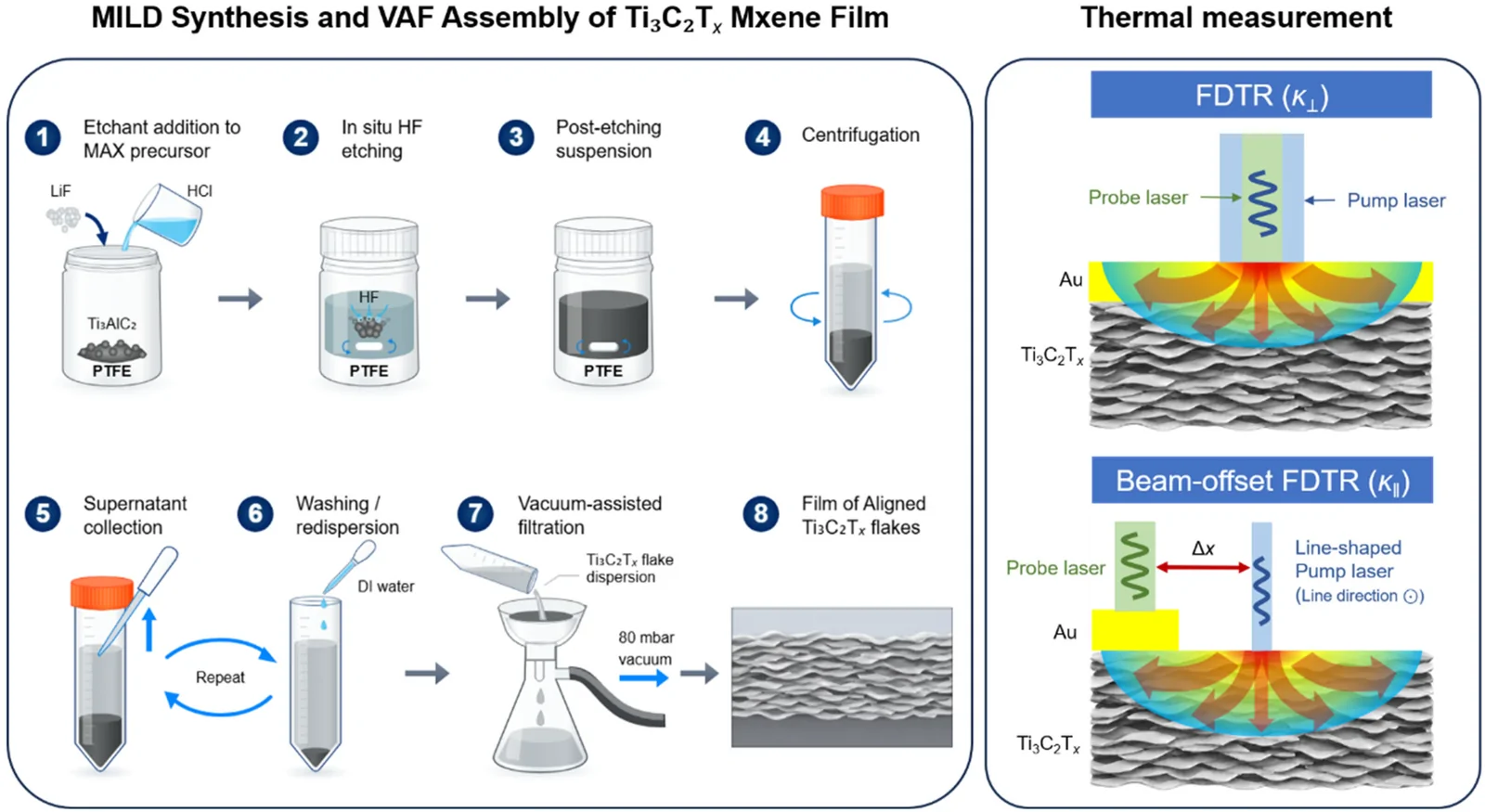
Preparation and thermal measurement configurations of Ti_3_C_2_T_*x*_ MXene films. The left panel illustrates the MILD synthesis and VAF assembly process. Ti_3_AlC_2_ MAX powder (0.75 g) was etched in a PTFE container using LiF (1.2 g) and 15 mL of 9 M HCl to generate HF *in situ*, followed by magnetic stirring at 750 rpm for 28 h at 35 °C. After etching, the suspension was centrifuged at 3500 rpm for 5 min and subjected to repeated washing/redispersion. The resulting dispersion was vacuum-filtered through a 100 nm-pore polycarbonate membrane at 80 mbar for 24 h to form an aligned Ti_3_C_2_T_*x*_ film. The upper right panel shows conventional FDTR for determining the cross-plane thermal conductivity, *κ*_⊥_, using a nominally 150 nm Au transducer. The lower right panel shows beam-offset FDTR for determining the in-plane thermal conductivity, *κ*_∥_, where a line-shaped pump heats a bare MXene region and the offset probe detects the response on an adjacent region coated with gold. The schematic represents a two-dimensional cross-section perpendicular to the long axis of the line-shaped pump, whose line direction points out of the page. Under this line-source geometry, no heat-flow component is depicted along the line direction, while the illustrated heat spreading emphasizes preferential in-plane transport parallel to the film surface.

This chemical etch method will lead to reproducible structural and compositional flakes with identical surface terminations mix, *i.e.* OH-, F- and O-functional groups. However, compared to aggressive hydrofluoric acid methods, the MILD route utilizes a water-rich, low-fluoride environment that may influence the coordination of titanium sites with ambient water molecules.^19–21^ While various functional groups coexist on the fully delaminated 2D basal planes, this process achieves a significant de-fluorination effect that systematically enriches the relative fraction of –OH terminations, thereby establishing a denser and more interconnected network of interlayer hydrogen bonds throughout the macroscopic assembly.^22^

### Characterization techniques

Powder X-ray diffraction (PXRD) patterns were collected on a Bruker D8 Advance A25 (DAVINCI) diffractometer operating at 40 kV and 40 mA with Cu Kα radiation (*λ* = 1.5419 Å). A 500 μm LYNXEYE XE energy-dispersive linear detector was used, optimized for high energy with the largest available aperture angle. Samples were mounted on monocrystalline silicon in powder form. The step size was 0.02° and the exposure time was 0.2 s per step. Field-emission scanning electron microscopy (FE-SEM) images were acquired using a Nova NanoSEM. Transmission electron microscopy (TEM) images were acquired using a JEOL 2100 microscope with holey carbon-coated Cu grids and Selected Area Electron Diffraction (SAED) recorded. The Raman spectra was characterized using a MonoVista CRS+ system (Spectroscopy and Imaging GmbH) under 532 nm laser excitation. A 50× objective focused the laser to a micro-spot, where the power was maintained at 0.5 mW to ensure non-destructive measurement. Spectra were recorded using a 30 s integration time sustained over 3 successive accumulations. Complementary diffraction gratings of 300 and 1500 lines per mm were implemented to capture both broad survey profiles and fine-structure spectral features.

### Thermal measurements

The thermal properties of the Ti_3_C_2_T_*x*_ films were characterized using Frequency Domain Thermoreflectance (FDTR),^23–25^ a high-resolution, non-contact optical technique that monitors the frequency-dependent phase response of a sample to periodic thermal excitation. In this setup, a sinusoidally modulated pump laser (*λ* = 445 nm, *P* = 12 mW) generated a periodic heat flux on the sample surface, with modulation frequencies sweeping from 100 kHz to 20 MHz. Simultaneously, a continuous-wave probe laser (488 nm, 6 mW) detected the resulting surface temperature fluctuations by measuring the thermoreflectance signal. To prevent sample degradation and minimize local heat accumulation, which is critical given the relatively low thermal conductivity of Ti_3_C_2_T_*x*_, strictly low laser powers were maintained throughout the measurements.

To ensure the accuracy of the heat conduction model, the thickness of the gold transducer layer was precisely calibrated prior to measuring the MXene films. A sapphire reference with well-established thermal properties was used as a standard sample for this calibration. For the conventional cross-plane configuration, a nominally 150 nm thick gold layer had been deposited onto both the sapphire reference and the MXene films *via* e-beam evaporation to act as a thermoreflectance transducer. By fitting the phase response of the Au/sapphire system (Fig. S3), the actual thickness of the gold layer was determined and subsequently treated as a fixed parameter during the thermal fitting of the Ti_3_C_2_T_*x*_ films. The experimental phase lag data were then fitted using an analytical 2D heat conduction model provided by Fourier Scientific LLC.^23^ In this fitting process, the *κ*_⊥_ and the thermal boundary conductance (TBC) at the gold/MXene interface were defined as the two independent free parameters. The volumetric specific heat of the films, essential for the FDTR model, was obtained using the reported specific heat,^26^ showing a value of 3.39 × 10^6^ J m^−3^ K^−1^ at room temperature. Other FDTR fitting processes, like sensitivity analysis, parameter set, *etc*., are illustrated in our SI.

To decouple the *κ*_∥_ from the out-of-plane components, we employed the beam-offset FDTR with 1D heat source.^27,28^ Under our experimental configuration, conventional concentric FDTR is primarily sensitive to *κ*_⊥_, whereas introducing a controlled spatial offset (*x*) between the pump and probe beams enhances sensitivity to in-plane heat transport (Fig. 1). We used an approximately one-dimensional line-shaped pump spot for heating, which substantially slowed the spatial decay of the thermal fluctuation signal with heat-transfer distance. This enabled measurements at larger pump–probe offsets, thereby further enhancing the sensitivity to in-plane heat transport and allowing the heat-transfer model to be simplified. Under this condition, the in-plane thermal diffusivity (*D*_∥_) can be extracted through a simple linear fitting procedure, as described by 
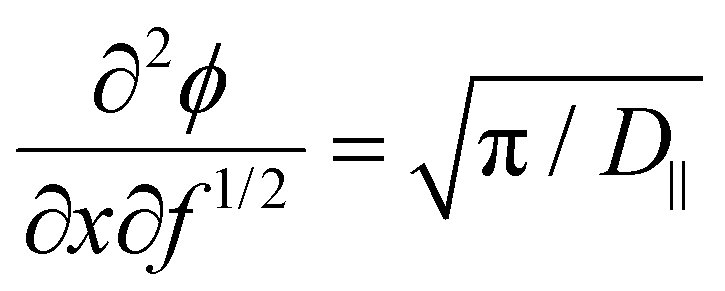
, where *ϕ* is the phase lag and *f* is the modulation frequency.^27^ Therefore, by scanning the phase response as a function of both modulation frequency and spatial offset, *D*_∥_ can be obtained directly without relying on additional input parameters. By combining the extracted in-plane thermal diffusivity with density and specific heat capacity data obtained from independent sources, the in-plane thermal conductivity can be calculated as *κ*_∥_ = *ρC*_p_*D*_∥_, where *ρ* is the mass density and *C*_p_ is the specific heat capacity.

## Results and discussion

Ti_3_C_2_T_*x*_ MXene flakes have been prepared following MILD method (Fig. 1) by etching and removing the aluminium present in MAX phase Ti_3_AlC_2_. The dark green supernatant demonstrating the Tyndall effect was observed by TEM (Fig. 2a). The acquired images reveal high-quality single and few-layer MXene flakes, which were further analyzed using electron diffraction. Samples observed are typically stored and aged under low temperature (down to 3 °C) and dark conditions over one month, pointing to the low degradation of our flakes over time under these storing conditions. In addition, hexagonal Selected Area Electron Diffraction (SAED) pattern proves the expected hexagonal crystalline structure of Ti_3_C_2_T_*x*_ MXene phase, where individual flakes are monocrystalline, in good agreement with the 2D structure of Ti_3_C_2_T_*x*_ MXene.^29^ Raman pattern (Fig. S2 in the SI) acts as a triple-check to clarify the quality synthesised single flake dispersion of Ti_3_C_2_T_*x*_.^30^ We observe the expected characteristic Raman bands in good agreement with previously reported data. We can detect peaks around 125 cm^−1^ and 205 cm^−1^ related to E_g_ and A1_g_ modes, respectively, those modes are attributed to vibrations of the Ti–C layer, in addition to 730 cm^−1^ signal (A1_g_) associated with C-related vibrations. A typical broad spectral features from 300 to 650 cm^−1^ is also detected recently assigned to mixed surface terminations, in accordance with our synthesis protocol, as shown in Fig. S2.

**Fig. 2:**
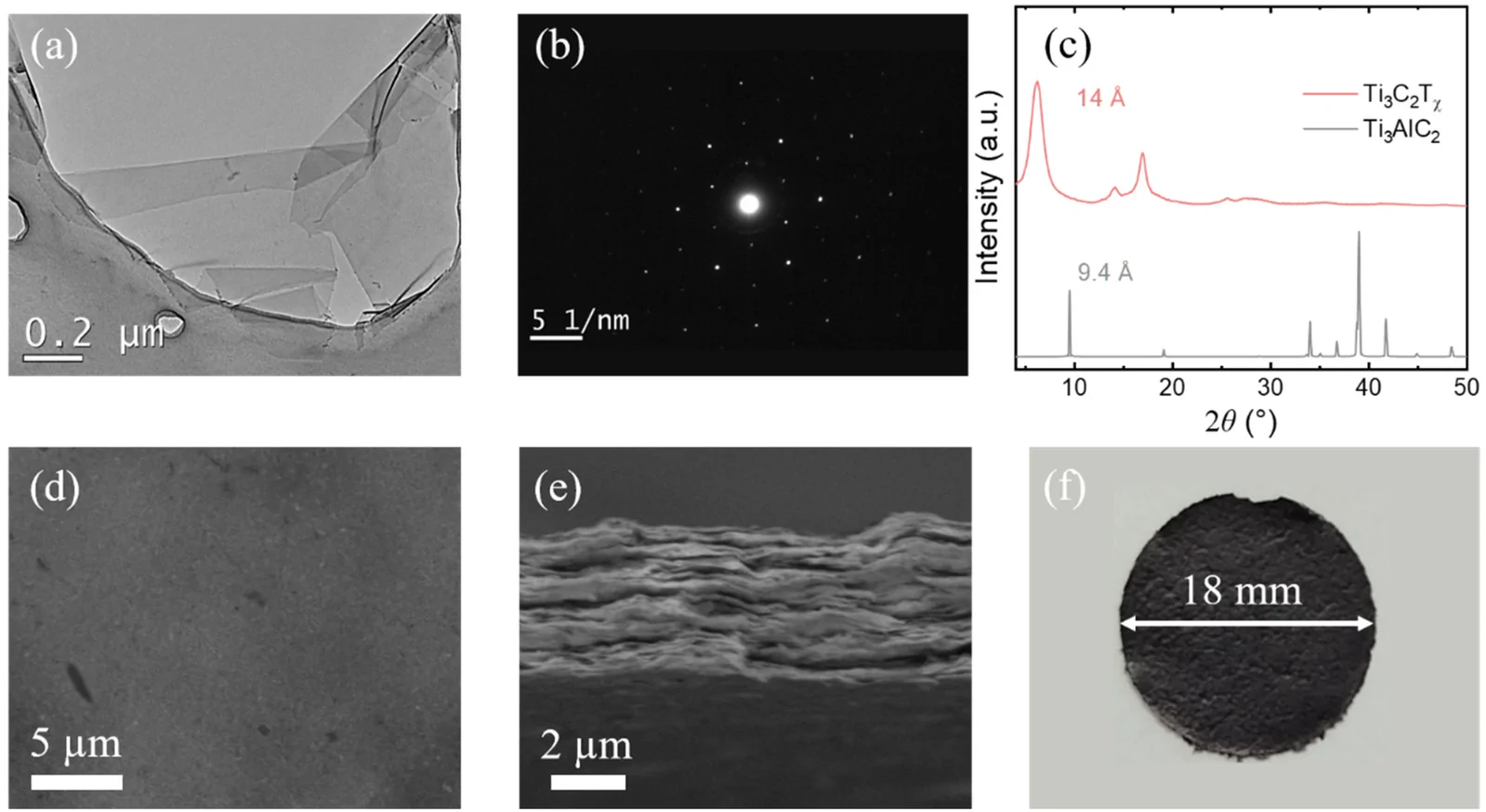
Morphological and structural characterization of Ti_3_C_2_T_*x*_ MXene flakes and films. (a) TEM image of a delaminated nanosheet. (b) Corresponding SAED pattern with hexagonal symmetry. (c) XRD patterns of the Ti_3_AlC_2_ precursor and Ti_3_C_2_T_*x*_ MXene; the marked interlayer spacings are 9.4 and 14 Å, respectively. (d) Top-view and (e) cross-sectional SEM images of the assembled film. (f) Photograph of a freestanding Ti_3_C_2_T_*x*_ film with a diameter of 18 mm.

MXene flakes in dispersion are ordered as continuous films assisted by vacuum filtration. X-ray diffraction collected on the formed films (Fig. 2c) confirms the ordering on the MXene layers showing a clear peak at low angle associated to the (002) plane and a second order (004), in addition of a smaller peak around 15° associated to the Polycarbonate film used as substrate. The flakes stacked forms a continuous freestanding film (Fig. 2f) with an oriented fashion of flakes. The surface picture shows the low roughness and very low to no presence of impurities such as halide salts. Also, neither porosity, wrinkles nor cracks are seen, highlighting the good mechanical assembly of the flakes (Fig. 2d). Despite direct drying over nitrogen flow that promotes the aggregation of rounded trend of aggregates after removing the water over nitrogen atmosphere, cross-sectional SEM images of the films (Fig. 2e) point that our films are around 5 μm thickness over preparations carried out at a pressure of 80 mbar in addition to the high orientation of the flakes. Taken together, the enhanced (00*l*) reflections and the lamellar morphology observed by cross-sectional SEM support preferential basal-plane alignment. However, because the present one-dimensional XRD configuration does not resolve the azimuthal orientation distribution, a quantitative Hermans orientation factor cannot be reliably extracted.

For the out-of-plane thermal measurement, to minimize errors stemming from local sample nonuniformity, thermal mapping was performed over a 100 × 100 μm^2^ area for each measurement, as illustrated in Fig. S5 (SI). Using a 1 μm step size, a total of 10 000 data points were collected per map to allow for robust statistical averaging. To ensure reliability and assess spatial uniformity, we performed six independent measurements across different regions of the sample (Table S1 in the SI). In the out of plane direction, the Ti_3_C_2_T_*x*_ films exhibit suppressed thermal conductivity. Extracting the *κ*_⊥_ from the high frequency phase response (Fig. 3a) yielded an average value of 0.54 ± 0.09 W m^−1^ K^−1^. This value is in line with 0.28 W m^−1^ K^−1^ for restacked films measured by a light flash system^35^ and 0.32 W m^−1^ K^−1^ for pure Ti_3_C_2_T_*x*_ MXene assemblies.^36^

**Fig. 3:**
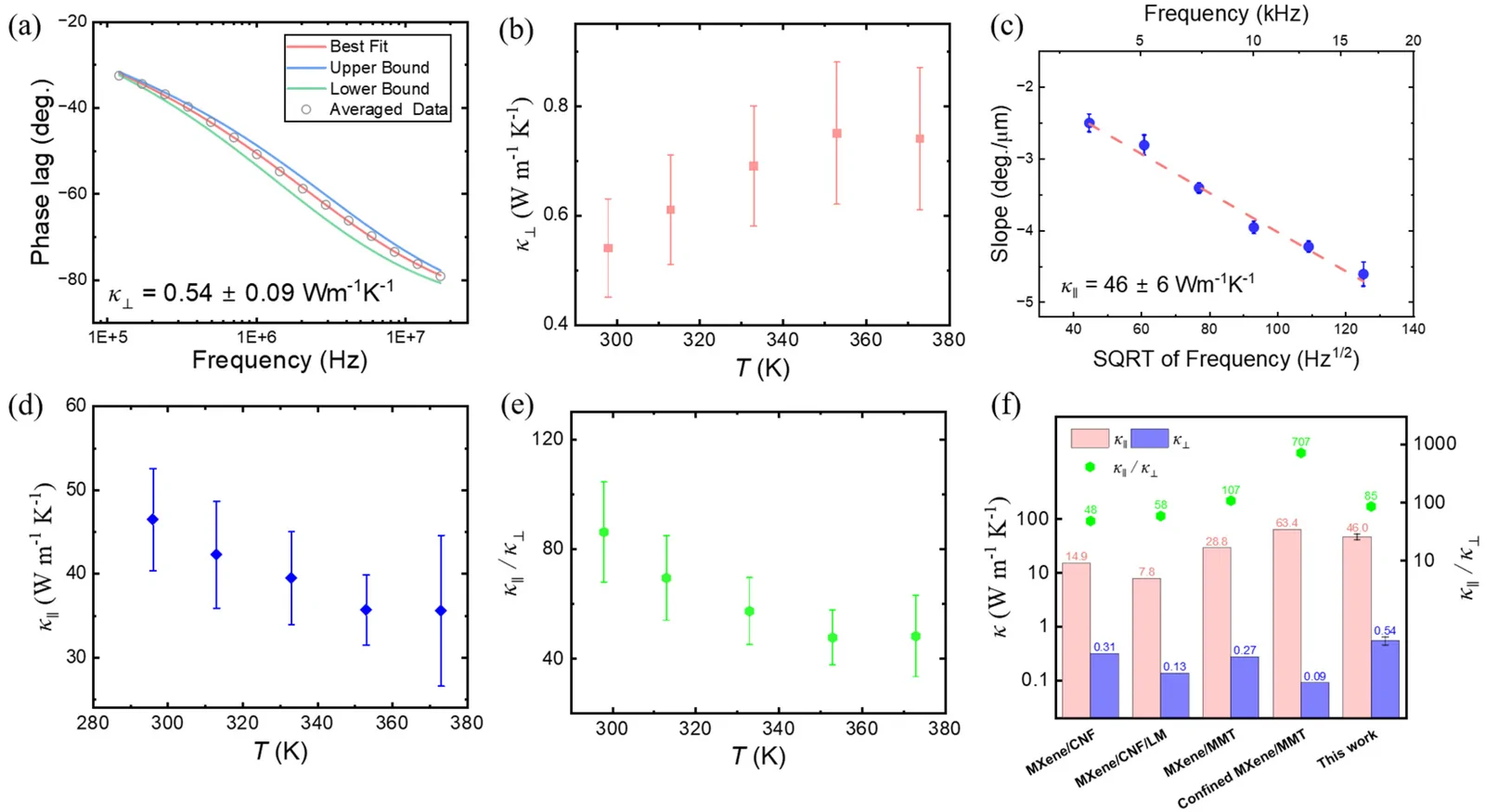
Thermal conductivity measurements of the Ti_3_C_2_T_*x*_ MXene film. (a) Experimental phase lag as a function of modulation frequency used to determine the cross-plane thermal conductivity (*κ*_⊥_). The open circles represent the averaged experimental data, while the solid red, blue, and green lines denote the best fit, upper bound, and lower bound of the fitting model, respectively. (b) Temperature-dependent out-of-plane thermal conductivity. (c) Slopes of phase *versus* pump–probe offset plotted against the square root of modulation frequency; the dashed fit gives an in-plane thermal conductivity (*κ*_∥_) of 46 ± 6 W m^−1^ K^−1^. (d) Temperature-dependent in-plane thermal conductivity. (e) Corresponding in-plane to out-of-plane thermal conductivity ratio. Error bars in panels b–e indicate the associated uncertainties. (f) Room-temperature comparison: pink and blue bars indicate in-plane and out-of-plane thermal conductivities on the left axis, and gray squares indicate their ratio on the right axis. From left to right, the reference films are MXene/CNF,^31^ MXene/CNF/liquid metal,^32^ MXene/MMT,^33^ and confined MXene/MMT,^34^ followed by this work. CNF denotes cellulose nanofibers and MMT denotes montmorillonite. LM denotes liquid metal, and MMT denotes montmorillonite. The displayed conductivities are rounded, while literature anisotropy ratios follow the reported or compiled values.

To investigate the underlying mechanism behind these observations, it is worth noting that employing the MILD processing may lead to a higher relative fraction of –OH terminations, which may modify cross-plane vibrational coupling compared with traditionally aggressive HF-etched counterparts.^19–21^ Microscopically, unlike the case of mixed terminations, a higher fraction of –OH groups may partly occupy the spatial voids between MXene layers, potentially increasing the interlayer atom density. In our VAF-assembled structure, –OH groups may form hydrogen-bonding networks with confined water molecules within the flake junctions.^22^ This may result in a filled interlayer interface that provides additional vibrational coupling paths across the van der Waals gaps.^37,38^ Consequently, such hydrogen bonding could contribute to the slight enhancement of our cross-plane thermal conductivity *κ*_⊥_ compared to previous reports. Nevertheless, even if local –OH-water networks lower the single-junction resistance, macro-scale transport remains strongly limited by the large number of interflake interfaces per unit thickness, preserving an exceptionally low *κ*_⊥_ in the out-of-plane direction.^39^

Furthermore, we evaluated the temperature dependence of the *κ*_⊥_ for the assembled film. The conductivity exhibits a positive temperature coefficient, increasing from 0.54 ± 0.09 W m^−1^ K^−1^ at 298 K to 0.75 ± 0.13 W m^−1^ K^−1^ at 353 K, before reaching a saturation plateau of 0.74 ± 0.13 W m^−1^ K^−1^ near 373 K, as shown in Fig. 3b. This observed positive trend mirrors the behavior of macroscopic delaminated films, yet it stands in stark contrast to the intrinsic behavior of individual Ti_3_C_2_T_*x*_ flakes. For isolated flakes, thermal conductivity exhibits a monotonic decline with temperature due to enhanced Umklapp scattering. For instance, theoretical calculations using the phonon Boltzmann transport equation for bare, fluorine-, and oxygen-terminated Ti_3_C_2_ monolayers show a continuous decrease: the thermal conductivity of Ti_3_C_2_ is 66.6 W m^−1^ K^−1^ at 300 K and drops to 35.4 W m^−1^ K^−1^ at 600 K. Conversely, in restacked Ti_3_C_2_T_*x*_ films, *κ*_∥_ rises from 1.26 W m^−1^ K^−1^ at 80 K to 2.84 W m^−1^ K^−1^ at 290 K,^40^ with some reports demonstrating enhancement up to 360 K.^41^ This contrast is consistent with an important role of thermal boundary conductance across physical interlayers. Increasing temperature may facilitate interflake vibrational energy transfer. The leveling near 373 K is consistent with a diminishing temperature dependence of effective interfacial transport, although the present data do not uniquely establish a microscopic saturation mechanism.

While the cross-plane direction acts as a significant bottleneck for heat propagation, the in-plane pathways within the same film exhibit drastically different transport kinetics. As shown in Fig. S4, the phase lag exhibits a characteristic linear decrease with increasing spatial offset between the pump and probe beams across a frequency range of 2.0 to 15.7 kHz. By extracting slopes from these profiles and plotting them against the square root of the modulation frequency (Fig. 3c), we obtained an *κ*_∥_ of 46 ± 6 W m^−1^ K^−1^ at room temperature. This relatively high value indicates efficient heat propagation along the 2D basal planes of the Ti_3_C_2_T_*x*_ flakes, facilitated by strong intra-layer covalent bonding and the high crystalline quality maintained by the MILD synthesis route. Together with the much lower *κ*_⊥_, this result is consistent with a preferentially aligned lamellar structure, in which heat can propagate more continuously along the basal planes while cross-plane transport is interrupted by repeated interflake interfaces. The enhanced (00*l*) reflections and lamellar morphology observed by cross-sectional SEM provide qualitative evidence for this structural picture, although the present measurements do not quantify the orientation distribution. Surface terminations and hydrogen bonding may further modify interfacial heat transfer.

For comparison, calculations for ideal Ti_3_C_2_ monolayers predict termination-dependent lattice thermal conductivities of about 108 and 11 W m^−1^ K^−1^ for fluorinated and oxygen-terminated structures, respectively.^16^ Individual-flake measurements have reported in-plane thermal conductivities of 30.6 to 105 W m^−1^ K^−1^ for approximately 9 to 45 nm thick flakes^42^ and 13 to 16 W m^−1^ K^−1^ using spatiotemporal microscopy.^43^ These intrinsic and individual-flake values should be distinguished from the effective thermal conductivity of assembled films, where interflake interfaces and structural alignment also influence heat transport. Our measured *κ*_∥_ of 46 ± 6 W m^−1^ K^−1^ is comparable to the 55.8 W m^−1^ K^−1^ reported for multilayered Ti_3_C_2_T_*x*_ architectures measured by Raman spectroscopy.^38^

The in-plane thermal conductivity decreases overall from approximately 46 W m^−1^ K^−1^ near room temperature to 35 W m^−1^ K^−1^ near 373 K (Fig. 3d). This behavior is consistent with enhanced phonon–phonon scattering at elevated temperatures, which reduces the phonon mean free path along the Ti_3_C_2_T_*x*_ basal planes, similar to the decreasing trend predicted for intrinsic Ti_3_C_2_-based layers^16^ and supported by recent measurements showing an important phonon contribution to in-plane heat transport in individual Ti_3_C_2_T_*x*_ flakes.^42,43^ In contrast, the cross-plane thermal conductivity increases with temperature, reflecting the stronger influence of repeated interflake interfaces, where increasing temperature can facilitate interfacial vibrational energy transfer. These different temperature dependences suggest distinct dominant transport mechanisms along the two directions and reduce the thermal anisotropy from approximately 85 to 47 over the measured temperature range (Fig. 3e).

Combining the in plane and out of plane measurements reveals a thermal anisotropy ratio of approximately 85. This metric provides a reliable benchmark for evaluating directional thermal management capabilities. While this value does not exceed the intrinsic anisotropy factor of high-quality bulk graphite, for which the cited benchmark is of the order of 100,^11^ it demonstrates substantial directional heat transport compared to various engineered two-dimensional composites. Within the class of MXene based structures, this performance surpasses the anisotropic factors reported for standard vacuum filtered architectures, including MXene/cellulose nanofiber films^44^ at 48 and MXene/cellulose nanofiber/liquid metal composites^45^ at approximately 58, as shown in Fig. 3f. Furthermore, the ratio of 85 closely matches the performance of complex electrostatically assembled networks, such as black phosphorus/boron nitride nanosheet/cellulose nanofiber films, which exhibit a ratio of 80.6.^46^ Although the anisotropy remains lower than that of highly oriented MXene/montmorillonite composites at 107 or extreme architectural designs,^36^ such as bioinspired confined alternating MXene/montmorillonite films reaching 707,^34^ achieving a ratio of 85 *via* minimally intensive layer delamination processing remains noteworthy. The observed ratio confirms that tuning intrinsic interlayer boundaries effectively balances transverse thermal insulation with competitive in plane heat spreading, avoiding the need for destructive thermal processing or complex hybrid integrations.

Driven by this anisotropic thermal behavior, these Ti_3_C_2_T_*x*_ films offer practical utility for advanced thermal management in miniaturized, high-power-density electronics. Specifically, the exceptionally low *κ*_⊥_ enables these films to function as effective thermal barriers that shield adjacent components from degradation, while the high anisotropy ratio of 85 allows them to act as electronic thermal spreaders that rapidly dissipate localized hotspots along the in-plane direction.^47^ Researchers have integrated similar anisotropic architectures into cooling systems for smartphones,^48^ high-power LED chips,^49^ and 5G printed circuit boards.^50^ Furthermore, such materials provide effective thermal management for energy devices; in lithium-ion battery packs, they dissipate operational heat while establishing a cross-plane thermal barrier to delay heat accumulation between adjacent cells.^51^ Combined with the intrinsic electrical conductivity of MXenes, these structurally aligned films suit multifunctional scenarios requiring both directional heat dissipation and electromagnetic interference shielding.^36^

## Conclusion

In conclusion, we have demonstrated an exceptionally high thermal anisotropy ratio of approximately 85 in Ti_3_C_2_T_*x*_ MXene films synthesized *via* the MILD method and assembled through VAF. Our synergistic measurements reveal that the *κ*_⊥_ is severely restricted to 0.54 ± 0.09 W m^−1^ K^−1^ at room temperature, whereas the in-plane pathways facilitate efficient heat propagation reaching 46 ± 6 W m^−1^ K^−1^. This anisotropy exceeds the CNF-containing MXene examples and is lower than the MMT-containing architectures in Fig. 3f, without requiring high temperature annealing. The temperature dependent analysis is consistent with an important role of the dense interlayer physical boundaries, resulting in a characteristic stabilization of thermal conductivity near 373 K. By successfully balancing competitive lateral heat spreading with effective transverse thermal insulation under MILD processing conditions, these highly oriented Ti_3_C_2_T_*x*_ films offer a versatile and high-performance platform for directional thermal management in miniaturized, high power density electronics. The overall decrease in in-plane conductivity with temperature further reduces the anisotropy to approximately 47 near 373 K.

## Author contributions

Wenkang Chen: investigation, formal analysis, visualization, writing – original draft, writing – review & editing. Mauro Ramos Jimenez: investigation, formal analysis, writing – original draft. Kai Xu: investigation, formal analysis, visualization, writing – review & editing. Maria-Anna Moustaka: investigation, formal analysis. Katja Klinar: writing – review & editing. Diego Collado Ramos: investigation, formal analysis, writing – review & editing. Xiangfan Xu: writing – review & editing, supervision. Jorge Rodríguez-Moreno: resources. Elena Navarrete-Astorga: resources, writing – review & editing, supervision. Alexandros El Sachat: resources, supervision. Javier Pérez Carvajal: investigation, formal analysis, visualization, writing – original draft, supervision. Miguel Muñoz Rojo: conceptualization, formal analysis, resources, writing – review & editing, funding acquisition, supervision.

## Conflicts of interest

There are no conflicts to declare.

## Supplementary Material

NR-OLF-D6NR02529C-s001

## Data Availability

The data supporting this article have been included as part of the supplementary information (SI). Supplementary information is available. See DOI: https://doi.org/10.1039/d6nr02529c.

## References

[cit1] Liu Y., Zou W., Zhao N., Xu J. (2023). Nat. Commun..

[cit2] Barani Z., Mohammadzadeh A., Geremew A., Huang C.-Y., Coleman D., Mangolini L., Kargar F., Balandin A. A. (2020). Adv. Funct. Mater..

[cit3] Chen J., Huang X., Zhu Y., Jiang P. (2017). Adv. Funct. Mater..

[cit4] Song S. H., Park K. H., Kim B. H., Choi Y. W., Jun G. H., Lee D. J., Kong B.-S., Paik K.-W., Jeon S. (2013). Adv. Mater..

[cit5] Shahil K. M. F., Balandin A. A. (2012). Nano Lett..

[cit6] Zeng X., Ye L., Yu S., Li H., Sun R., Xu J., Wong C.-P. (2015). Nanoscale.

[cit7] Zeng X., Yao Y., Gong Z., Wang F., Sun R., Xu J., Wong C.-P. (2015). Small.

[cit8] Madeshwaran S. R., Jayaganthan R., Velmurugan R., Gupta N. K., Manzhirov A. V. (2018). J. Phys.: Conf. Ser..

[cit9] Zhou K., Liu J., Zeng W., Hu Y., Gui Z. (2015). Compos. Sci. Technol..

[cit10] Kim S. E., Mujid F., Rai A., Eriksson F., Suh J., Poddar P., Ray A., Park C., Fransson E., Zhong Y., Muller D. A., Erhart P., Cahill D. G., Park J. (2021). Nature.

[cit11] Renteria J. D., Ramirez S., Malekpour H., Alonso B., Centeno A., Zurutuza A., Cocemasov A. I., Nika D. L., Balandin A. A. (2015). Adv. Funct. Mater..

[cit12] Qian X., Zhou J., Chen G. (2021). Nat. Mater..

[cit13] Naguib M., Mochalin V. N., Barsoum M. W., Gogotsi Y. (2014). Adv. Mater..

[cit14] Shahzad F., Alhabeb M., Hatter C. B., Anasori B., Hong S. M., Koo C. M., Gogotsi Y. (2016). Science.

[cit15] Lukatskaya M. R., Kota S., Lin Z., Zhao M.-Q., Shpigel N., Levi M. D., Halim J., Taberna P.-L., Barsoum M. W., Simon P., Gogotsi Y. (2017). Nat. Energy.

[cit16] Gholivand H., Fuladi S., Hemmat Z., Salehi-Khojin A., Khalili-Araghi F. (2019). J. Appl. Phys..

[cit17] Mani D., Vu M. C., Lim C.-S., Kim J.-B., Jeong T.-H., Kim H. J., Islam M. A., Lim J.-H., Kim K.-M., Kim S.-R. (2023). Carbon.

[cit18] Anjum N., Rahman M. M., Elattar A., Sijuade A., Kalu E. E., Okoli O. I. (2025). Adv. Sustainable Syst..

[cit19] Alhabeb M., Maleski K., Anasori B., Lelyukh P., Clark L., Sin S., Gogotsi Y. (2017). Chem. Mater..

[cit20] Kobayashi T., Sun Y., Prenger K., Jiang D., Naguib M., Pruski M. (2020). J. Phys. Chem. C.

[cit21] Nouseen S., Pumera M. (2025). J. Mater. Chem. A.

[cit22] Lounasvuori M., Zhang T., Gogotsi Y., Petit T. (2024). J. Phys. Chem. C.

[cit23] Schmidt A. J., Cheaito R., Chiesa M. (2009). Rev. Sci. Instrum..

[cit24] Schmidt A., Chiesa M., Chen X., Chen G. (2008). Rev. Sci. Instrum..

[cit25] Schmidt A. J., Chen X., Chen G. (2008). Rev. Sci. Instrum..

[cit26] Srivatsa S., Tokarz W., Przewoźnik J., Strączek T., Grabowski K., Rutkowski P., Uhl T., Kulawik J., Kata D., Madej D., Lis J., Kapusta C. (2024). Materials.

[cit27] Xu K., Guo J., Raciti G., Goni A. R., Alonso M. I., Borrisé X., Zardo I., Campoy-Quiles M., Reparaz J. S. (2023). Int. J. Heat Mass Transfer.

[cit28] Xu K., Skorda S., Xiao P., Coy E., Cartoixà X., Rurali R., Reparaz J. S., El Sachat A. (2025). Nano Lett..

[cit29] Ahmed B., Anjum D. H., Hedhili M. N., Gogotsi Y., Alshareef H. N. (2016). Nanoscale.

[cit30] Furchner A., Parker T., Mauchamp V., Hurand S., Plaickner J., Rappich J., Emerenciano A. A., Hinrichs K., Gogotsi Y., Petit T. (2025). J. Phys. Chem. C.

[cit31] Jiao E., Wu K., Liu Y., Lu M., Hu Z., Chen B., Shi J., Lu M. (2021). Composites, Part A.

[cit32] Ran L., Ma X., Qiu L., Sun F., Zhao L., Yi L., Ji X. (2023). J. Colloid Interface Sci..

[cit33] Li L., Cao Y., Liu X., Wang J., Yang Y., Wang W. (2020). ACS Appl. Mater. Interfaces.

[cit34] Li L., Xu R., Yan J., Wang Y., Lu J., Zhang Z., Li W., Lian W., Sun J., Jia B., Cheng Q. (2026). Adv. Funct. Mater..

[cit35] Li L., Shi M., Liu X., Jin X., Cao Y., Yang Y., Wang W., Wang J. (2021). Adv. Funct. Mater..

[cit36] Li L., Cao Y., Liu X., Wang J., Yang Y., Wang W. (2020). ACS Appl. Mater. Interfaces.

[cit37] Li J., Chi Z., Qin R., Yan L., Lin X., Hu M., Shan G., Chen H., Weng Y.-X. (2020). J. Phys. Chem. C.

[cit38] Hu T., Hu M., Li Z., Zhang H., Zhang C., Wang J., Wang X. (2016). Phys. Chem. Chem. Phys..

[cit39] Liu Y., Wu Y., Wang X. (2024). Nano Res..

[cit40] Chen L., Shi X., Yu N., Zhang X., Du X., Lin J. (2018). Materials.

[cit41] Wan Z., Li C., Liu R., Zhou W., Fan W., Huang C., Liu Y. (2024). ACS Appl. Mater. Interfaces.

[cit42] Liu Y., Rahbar M., Karamati A., Fei Z., Chi Y., Snyder G. J., Isotta E., Wang X., Wu Y. (2026). Commun. Mater..

[cit43] van Hemert M. C., Ramsden H., Ippolito S., Varghese S., Liu B., Tornero Gasca E., Muñoz Rojo M., Wang H. I., Gogotsi Y., Tielrooij K.-J. (2026). Nano Lett..

[cit44] Jiao E., Wu K., Liu Y., Lu M., Hu Z., Chen B., Shi J., Lu M. (2021). Composites, Part A.

[cit45] Ran L., Ma X., Qiu L., Sun F., Zhao L., Yi L., Ji X. (2023). J. Colloid Interface Sci..

[cit46] Wang Y., Wei X., Cai H., Zhang B., Chen Y., Li M., Qin Y., Li L., Kong X., Gong P. (2022). Nanoscale.

[cit47] Li L., Cheng Q. (2024). Interdiscip. Mater..

[cit48] Chen Q., Ma Z., Wang Z., Liu L., Zhu M., Lei W., Song P. (2022). Adv. Funct. Mater..

[cit49] He X., Zhang K., Wang H., Zhang Y., Xiao G., Niu H., Yao Y. (2022). Carbon.

[cit50] Chen Y., Zhang H., Chen J., Guo Y., Jiang P., Gao F., Bao H., Huang X. (2022). ACS Nano.

[cit51] Guo H., Zhao H., Niu H., Ren Y., Fang H., Fang X., Lv R., Maqbool M., Bai S. (2021). ACS Nano.

