# Supplementary material for: Highly aligned Ti3C2Tx MXene films with high anisotropic thermal conductivity
Source: Nanoscale. 2026 Oct 19. Online ahead of print. doi: 10.1039/d6nr02529c (PMC13712111; doi:10.1039/d6nr02529c)
Supplement: Supplementary file 1 [file NR-OLF-D6NR02529C-s001.pdf]

Supplementary Information (SI) for Nanoscale.
This journal is © The Royal Society of Chemistry

Highly Aligned $Ti_3C_2T_x$ MXene Films with High Anisotropic Thermal Conductivity

Wenkang Chen[1,2,*], Mauro Ramos Jimenez[1], Kai Xu[3], Marianna Moustaka[3], Katja Klinar[4], Diego Collado Ramos[1], Xiangfan Xu[2], Jorge Rodríguez Moreno[5], Elena Navarrete Astorga[6], Alexandros El Sachat[3], Javier Pérez Carvajal[1], Miguel Muñoz Rojo[1,7,‡]

1) Institute of Material Science of Madrid - CSIC, 28049 Madrid (Spain)
2) Center for Phononics and Thermal Energy Science, China-EU Joint Center for Nanophononics, School of Physics Science and Engineering, Tongji University, 200092 Shanghai (China)
3) Institute of Nanoscience and Nanotechnology, National Center for Scientific Research "Demokritos," Athens, Agia Paraskevi 15341, Greece
4) University of Ljubljana, Faculty of Mechanical Engineering, Laboratory for refrigeration and district energy, Askerceva 6, 1000 Ljubljana (Slovenia)
5) Electronic Technology Department, University of Malaga, 29071 Málaga (Spain)
6) Materials and Surfaces Laboratory, Applied Physics I Department, University of Málaga, 29071 Málaga (Spain)
7) Department of Thermal and Fluid Engineering, University of Twente, Enschede (Netherlands)
‡ m.m.rojo@csic.es

S1 Film preparation and characterization

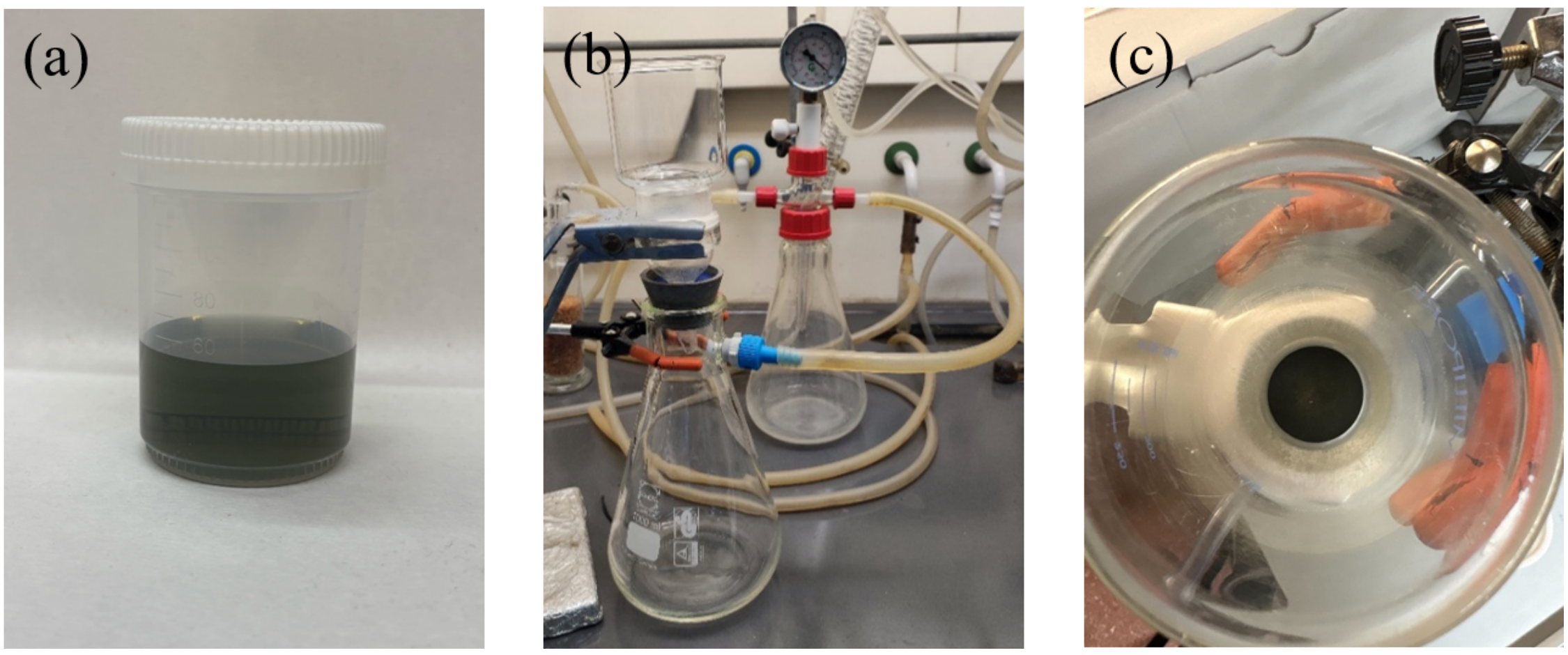

Figure S1. (a) Optical image of the stable $Ti_3C_2T_x$ MXene colloidal suspension, exhibiting a characteristic dark green coloration indicative of well-delaminated individual or few-layer flakes. (b) Experimental setup employed for the vacuum-assisted filtration process. (c) Top-down view of the filtration funnel during process, demonstrating the uniform deposition and accumulation of $Ti_3C_2T_x$ flakes on the porous membrane filter.

To prepare a freestanding $Ti_3C_2T_x$ MXene film via the vacuum-assisted filtration method, a stable aqueous MXene suspension was first synthesized following the minimally intensive layer delamination (MILD) etching protocol from the parent Ti₃AlC₂ MAX phase. As shown in Figure S1a, a homogeneous dark green suspension was obtained after redispersing the dark black MXene slurry recovered from the final centrifugation step. The well-dispersed suspension, which appeared visually translucent and free of macroscopic aggregations under ambient light, was subsequently poured onto a membrane filter housed within the vacuum filtration apparatus (Figure S1b). Upon application of a continuous vacuum, the solvent permeated through the porous membrane while the delaminated $Ti_3C_2T_x$ flakes were intercepted at the surface, gradually constructing a thin, uniform, and highly aligned film architecture (Figure S1c). Following complete filtration, the wet film was dried under ambient temperature to thoroughly eliminate residual water molecules. Finally, the consolidated $Ti_3C_2T_x$ MXene film was carefully peeled off from the substrate to yield a flexible, freestanding thermal barrier membrane.

S2 Raman characterization

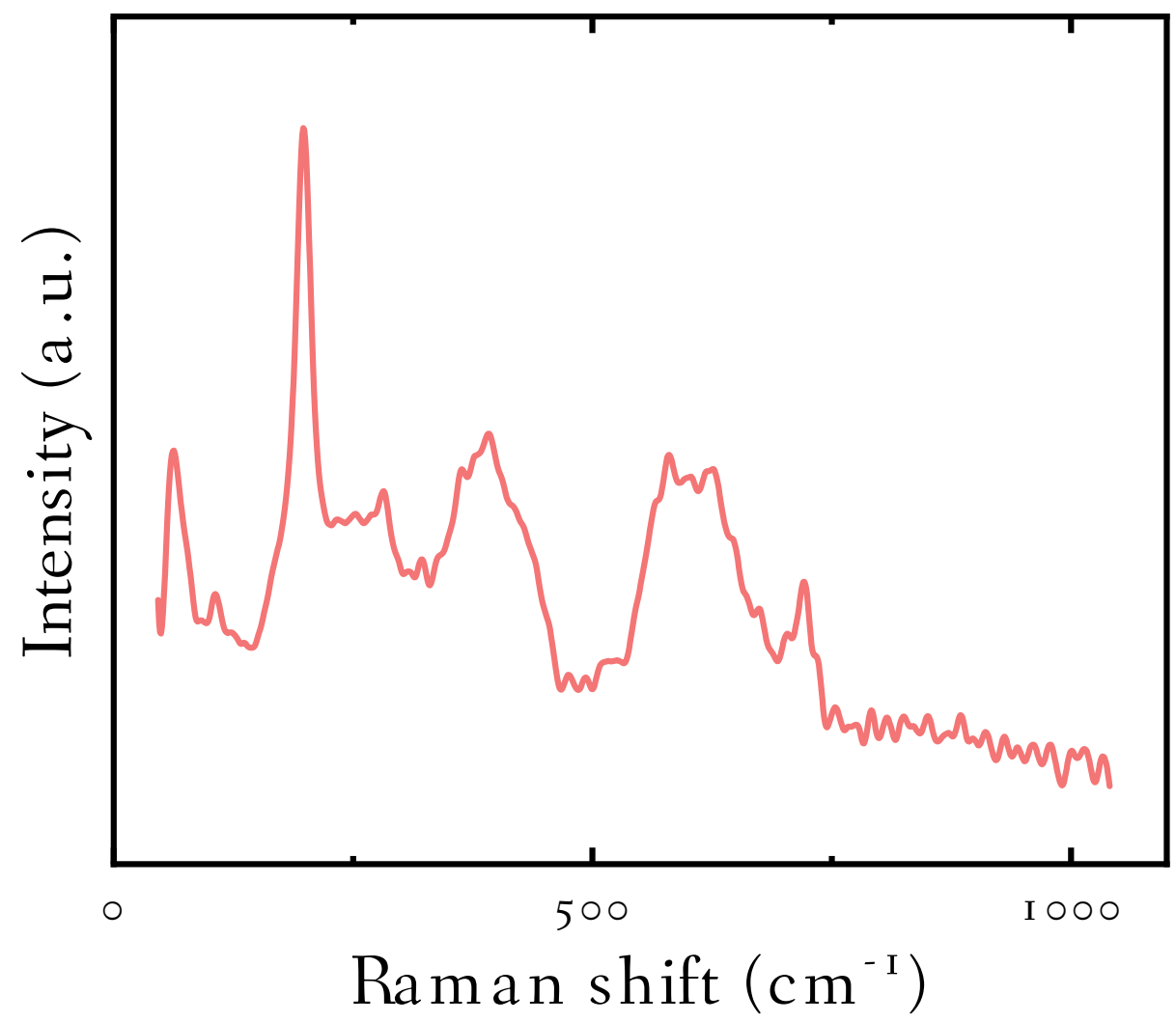

Figure S2 Raman spectroscopy of $Ti_3C_2T_x$ film. Peaks around 125 cm⁻^{1} and 205 cm⁻^{1} correspond to Eg and A1g modes, respectively, which are attributed to vibrations of the Ti–C layer. Additionally, a signal at 730 cm⁻^{1} (A1g) is associated with C-related vibrations. Typical broad spectral features from 300 to 650 cm⁻^{1}, assigned to mixed surface terminations, are also detected, consistent with our synthesis protocol.

S3 Gold thickness calibration on Sapphire

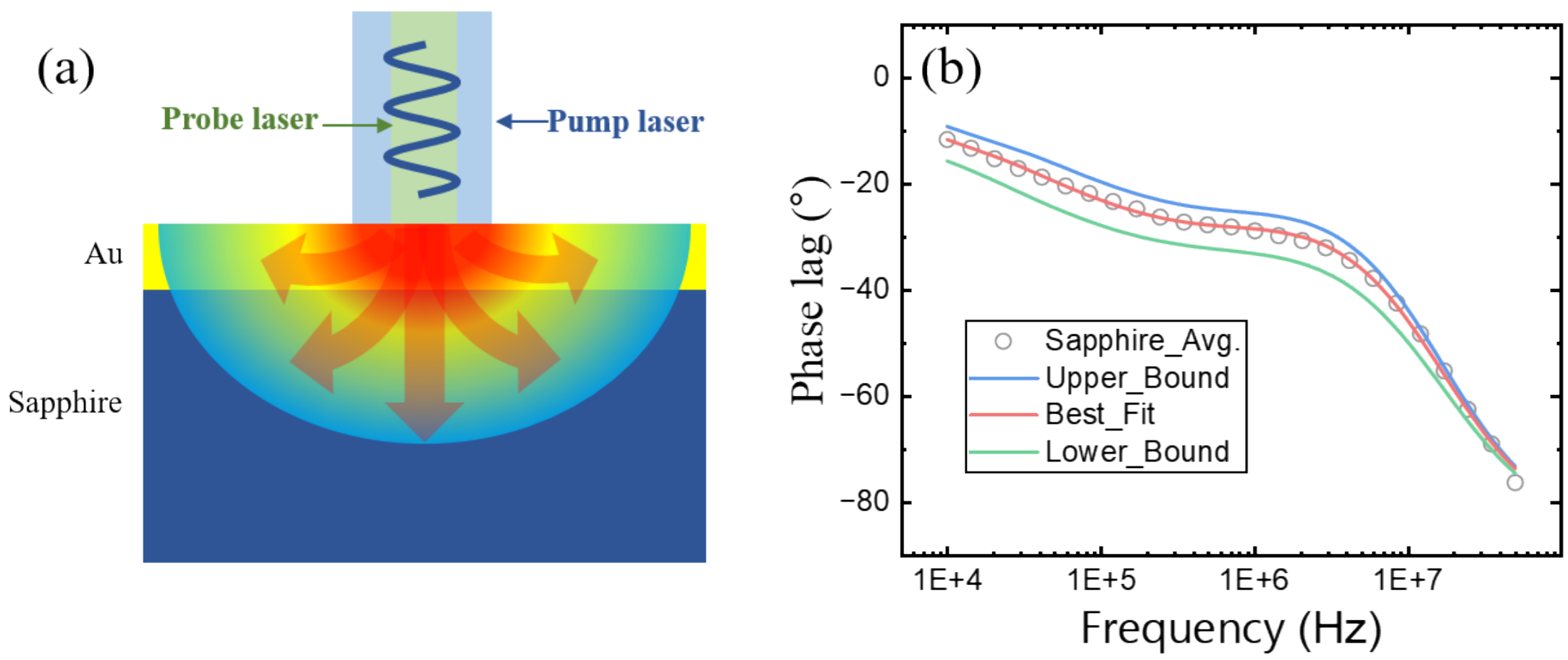

Figure S3 Gold transducer thickness calibration using the sapphire reference sample. (a) Schematic of the conventional FDTR measurement on the Au coated sapphire reference. (b) Experimental phase lag as a function of modulation frequency together with the best fit and the corresponding upper and lower fitting bounds. The fitted Au thickness was used as a fixed parameter in the subsequent FDTR analysis of the $Ti_3C_2T_x$ films.

Before the thermal measurements of the $Ti_3C_2T_x$ films, the Au transducer thickness was calibrated using an Au coated sapphire reference sample prepared in the same e beam evaporation process. The measured FDTR phase response was fitted using the known thermal properties of sapphire, with the Au thickness treated as the fitting parameter. As shown in Figure S3b, the experimental phase data agree well with the best fit, while the upper and lower bounds indicate the fitting uncertainty. The

calibrated Au thickness (150 nm) was subsequently used as a fixed input parameter in the thermal analysis of the $Ti_3C_2T_x$ films.

S4 In-plane thermal measurement

To illustrate the beam-offset data used to extract the in-plane thermal conductivity, Figure S4 shows the measured phase lag as a function of pump–probe offset at six modulation frequencies between 2.0 and 15.7 kHz. At each frequency, the phase lag decreases approximately linearly with offset, and the magnitude of the fitted slope increases with modulation frequency. These fitted slopes were subsequently used in the analysis shown in Figure 3c of the main manuscript.

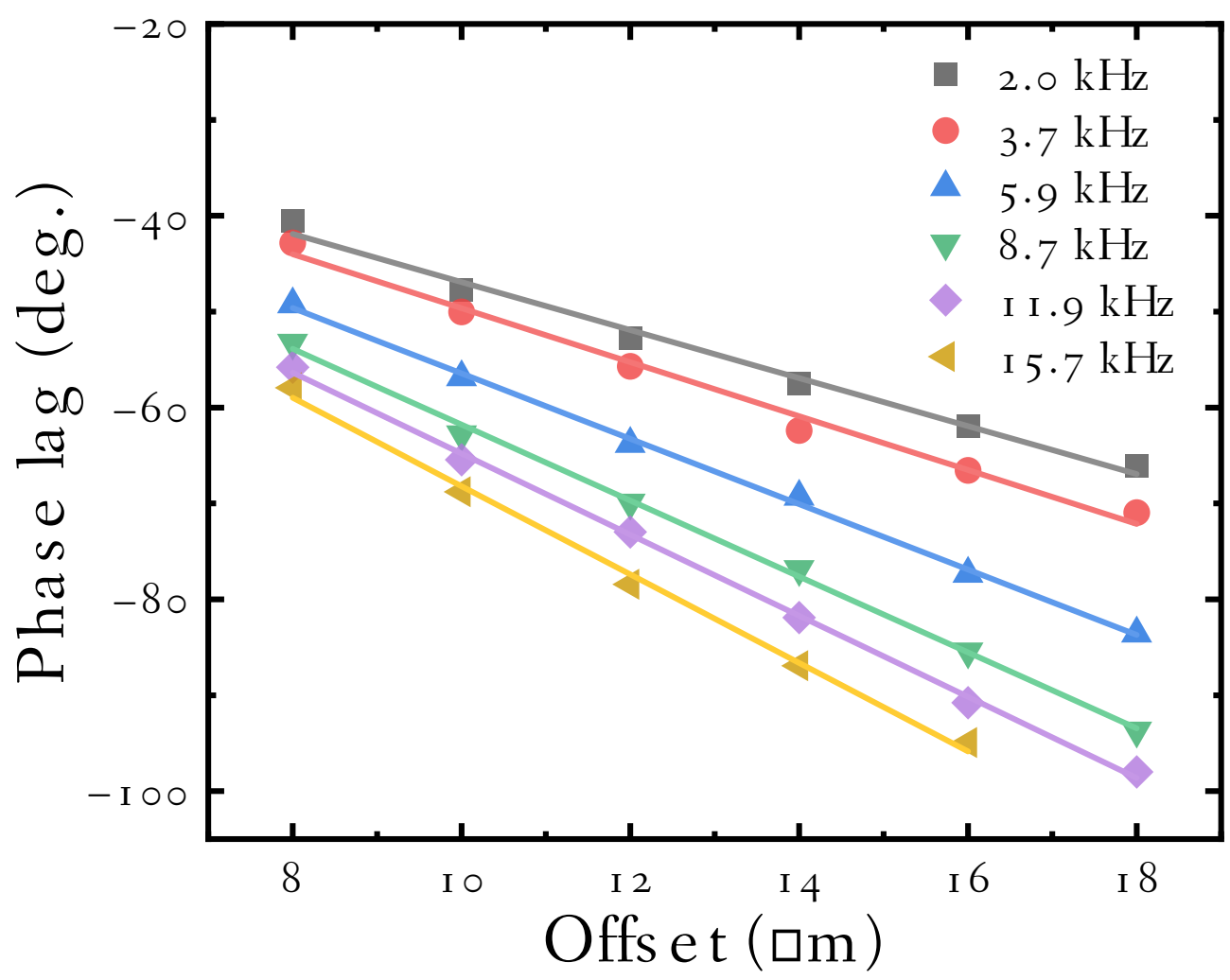

Figure S4. Offset-dependent phase responses of the $Ti_3C_2T_x$ film measured by beam-offset FDTR at modulation frequencies of 2.0, 3.7, 5.9, 8.7, 11.9, and 15.7 kHz. Symbols represent the measured phase lag, and solid lines show linear fits over the 8–18 μm offset range. The fitted slopes were used to determine the in-plane thermal diffusivity and thermal conductivity.

S5 Thermal map by frequency domain thermoreflectance (FDTR)

We characterize the thermal properties of $Ti_3C_2T_x$ by FDTR technique.[1–5] The principle of this technique and the detail of experimental operation has been illustrated in the main text. We performed thermal mapping over a 100 × 100 μm^2 area with 10,000 sampling points. The thermal map of FDTR typically includes two channels: the amplitude channel and the phase channel, as shown in Figure S5a and S5b. These two maps show relatively uniform signals at 2.05 × 10⁶ Hz for both channels, confirming a good quality of our $Ti_3C_2T_x$ film samples.

Fundamentally, the modulated pump beam induces a complex thermal response demodulated by a lock-in amplifier into orthogonal amplitude and phase components. The amplitude channel records the absolute oscillation intensity, which is highly sensitive to surface topography and optical alignment, serving as a diagnostic tool for mapping uniformity. Conversely, the phase channel measures the phase lag between the heat flux and temperature response. Because the phase is resolved from the ratio of the imaginary to real signal components, it inherently cancels absolute fluctuations in laser power,

alignment, and surface roughness, providing an artifact-free metric that directly reflects the intrinsic thermal properties of the material stack. Every pixel contains a phase versus frequency signal. The final

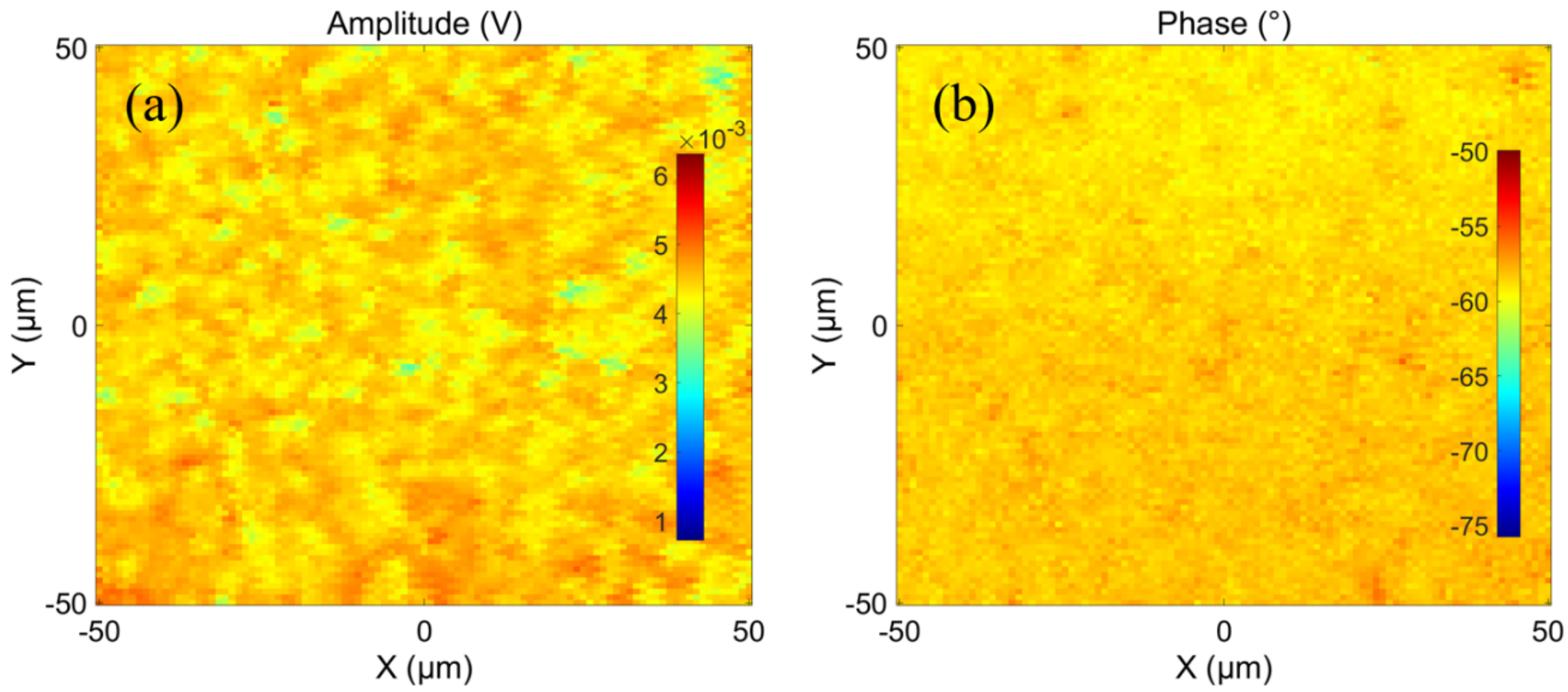

Figure S5 Thermal maps at 2.05 × 10⁶ Hz by frequency domain thermoreflectance. (a) amplitude channel and (b) phase channel.

signal was acquired by averaging over the entire map, as shown in Figure 3a in the main text, from which the out-of-plane thermal conductivity(□) can be extracted based on the multilayer heat transfer model detailed below.

S6 Thermal transport modeling and data reduction

The heat transfer model for FDTR analytically solves the heat diffusion equation for a periodically modulated Gaussian pump laser heating a multilayer material stack.[4] The model employs a frequency-domain matrix formulation to compute layer-by-layer heat flow, integrating specific parameters including thickness, volumetric heat capacity, anisotropic thermal conductivities, and thermal boundary conductance.[5] To determine the theoretical thermal response, the model applies an inverse Hankel transform to the calculated temperature distribution and weights the result by the Gaussian

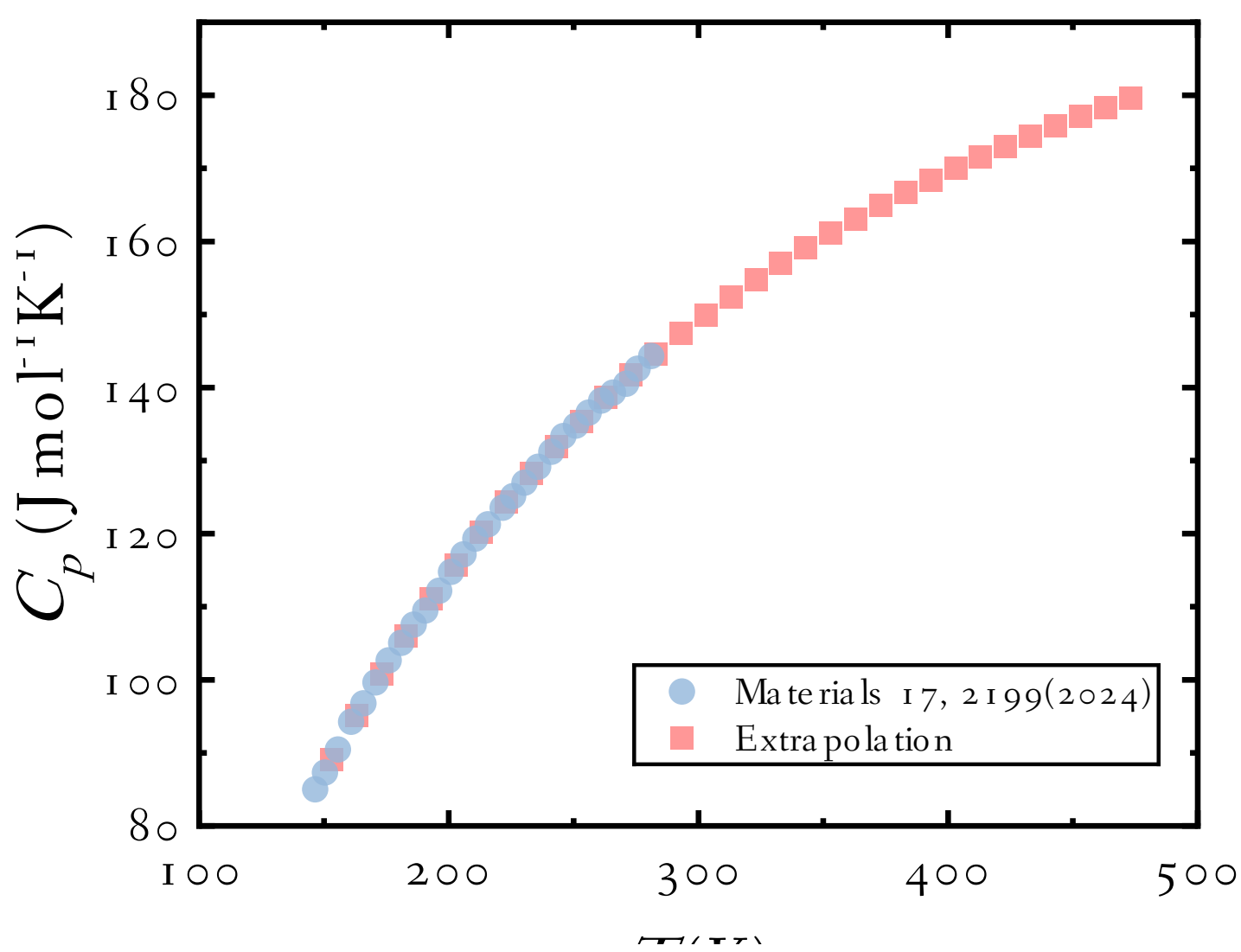

Figure S6 Specific heat from literature and extrapolation based on Maier-Kelley formulation

intensity profile of the probe beam.[1] Then compare this analytical solution against experimental phase data using a least-squares minimization algorithm to extract the unknown thermal properties of the sample.[1]

Here, we applied a two-layer model to fit our phase versus frequency data. The first layer is gold with 150 nm in thickness, and the thermal conductivity and specific heat are taken from literature. The second layer is MXene film with 5 μm in thickness, and its in-plane thermal conductivity is set to be 46 W m⁻^{1} K⁻^{1}. $□_{□}$ and the TBC are chosen as the two fitting inputs. As for the specific heat, we applied the molar heat capacity from literature,[6] which is around 147.4 J mol⁻^{1} K⁻^{1} at room temperature. Thus, the volumetric specific heat (C_v) is calculated to be 3.39 × 10⁶ J m⁻^{3} K⁻^{1} at room temperature by:

$$C_v = C_p \times \frac{ρ}{M} \tag{1}$$

Where M is the molar mass of Ti₃C₂OH (184.6 g mol⁻^{1}) and ρ is the density, which is 4.21 g cm⁻^{3} at room temperature. [7] We also did extrapolation to get the specific heat for the high temperature measurements, as shown in Figure S6. We applied Maier-Kelley formulation to do the extrapolation[8]:

$$C_p = a - bT^{-1} - cT^{-2} \tag{2}$$

From a solid-state physics perspective, this mathematical framework inherently satisfies the harmonic approximation of lattice dynamics. As temperature rises and high-frequency phonon modes approach complete excitation, the heat capacity growth rate (dC_p/dT) monotonically decelerates, gradually converging toward a finite asymptotic limit. This boundary condition aligns with the theoretical Dulong-Petit behavior at elevated temperatures,[9] thereby ensuring a mathematically stable and physically plausible extrapolation up to 473 K.

S7 Sensitivity and uncertainty of FDTR model

To substantiate the accuracy, uniqueness, and statistical rigor of the multi-parameter estimation routine utilized in our cross-plane frequency-domain thermoreflectance data reduction, a systematic validation

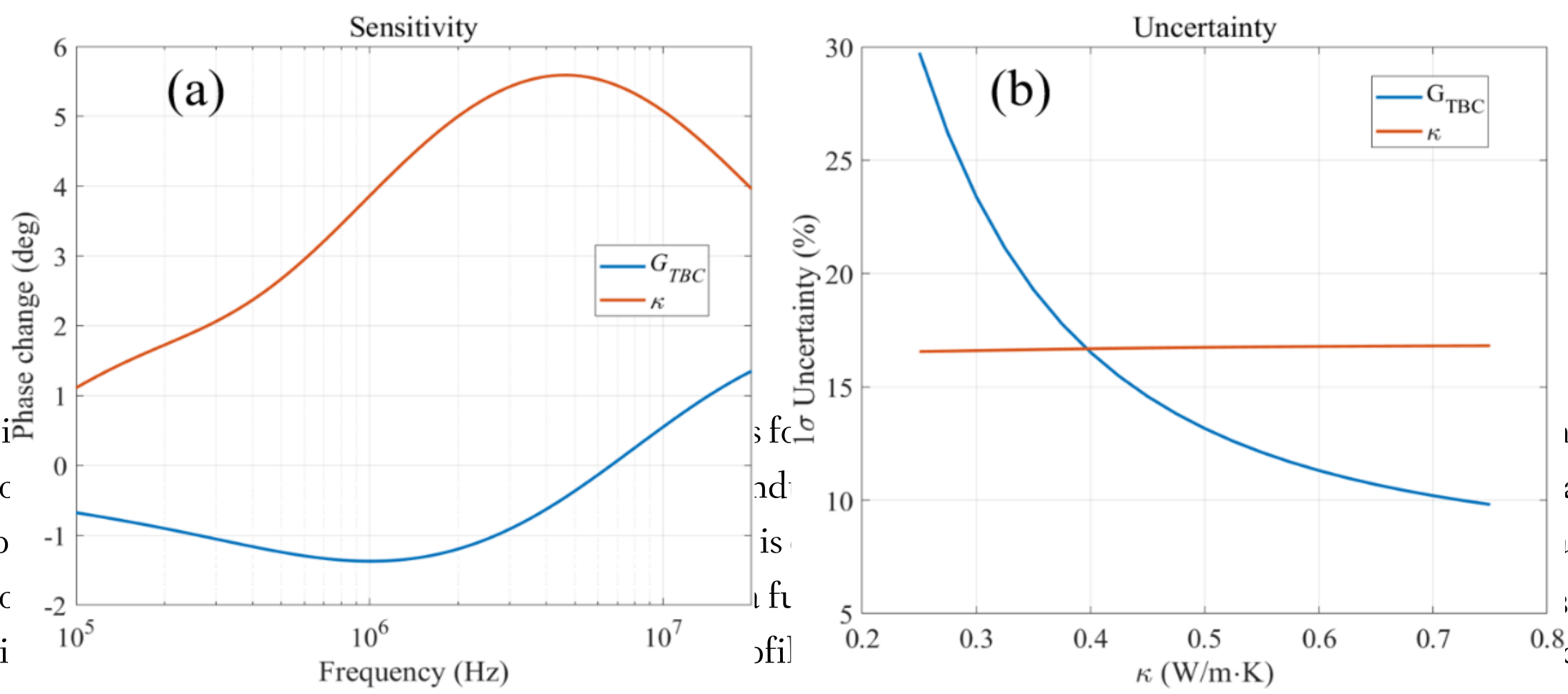

evaluating phase sensitivities, error propagation, and parameter boundaries was conducted. The following descriptions outline the analytical significance of the two diagnostic plots presented in Figure S7.

To ensure the fidelity and uniqueness of the multi-parameter estimation routine, the orthogonality of the primary fitting parameters was rigorously verified through frequency-dependent phase sensitivity analysis (Figure S7a). The derivative profile for the □$_{□}$ of the MXene film exhibits a robust, high-magnitude peak exceeding 5° near a modulation frequency of 4.5 × 10⁶ Hz. In sharp contrast, the sensitivity trajectory of the gold/MXene G_TBC remains suppressed within this optimal frequency window, reaching a local minimum of -1.4° near 10⁶ Hz and traversing toward a modest positive response only at frequencies exceeding 5 × 10⁶ Hz. This pronounced divergence in both mathematical profile and peak frequency effectively eliminates critical parameter correlation, guaranteeing that □$_{□}$ can be extracted independently with high precision.

The statistical rigor and measurement reliability are further substantiated by error propagation analysis across a broad, physically relevant trial range of 0.25 to 0.75 W m⁻^{1} K⁻^{1} (Figure S7b). Across this entire domain, the propagated 1σ uncertainty for □$_{□}$ remains remarkably flat and tightly bounded between 16.5% and 17%. Concurrently, the uncertainty profile for the interface G_TBC systematically decays from 30% and stabilizes below 10% at higher thermal conductivity values, intersecting the □$_{□}$ uncertainty curve at approximately 0.4 W m⁻^{1} K⁻^{1}. This clean error convergence and the low, invariant baseline of the □$_{□}$ uncertainty curve demonstrate that the extracted thermal properties are highly resilient against potential experimental phase noise, confirming the exceptional reproducibility of our methodology.

The experimental phase lag trajectory, sweeping from 10⁵ Hz to 2 × 10⁷ Hz, is symmetrically and tightly bracketed by the simulated upper and lower boundary curves representing controlled parameter perturbations, as shown in Figure 3a. The non-divergent encapsulation of the phase data within these narrow envelopes confirms that the physical model establishes an exceptionally stable optimization landscape for unique parameter extraction.

S8 Statistics of out-of-plane thermal conductivity on different regions

To verify the macroscopic spatial uniformity and reproducible thermal performance of the fabricated freestanding $Ti_3C_2T_x$ MXene films, systematic frequency-domain thermoreflectance (FDTR) measurements were executed across six randomly selected regions. The localized statistical results are summarized in Table S1. The determined □$_{□}$ exhibits an exceptionally narrow distribution, yields a tightly constrained mean value of 0.54 W m⁻^{1} K⁻^{1}, and maintains a remarkably low region-to-region standard deviation of only 0.04 W m⁻^{1} K⁻^{1}. Concurrently, the interfacial G_{TBC} stabilizes consistently within the identical order of magnitude, centering at a mean value of 1.36 × 10⁷ W m⁻^{2} K⁻^{1}. This high degree of spatial consistency and minimal data scattering across disparate zones rigorously demonstrate the excellent structural homogeneity of our vacuum-filtered MXene assemblies, confirming that the directional phonon scattering engineered at the dense interlayer boundaries is uniform and highly reproducible throughout the entire macroscopic film network.

Table S1 Statistics of out-of-plane thermal conductivity(κ⊥) and thermal boundary conductance on different regions

Region	TBC (W m^{-2} K^{-1})	Abs Error (TBC)	κ⊥ (W m^{-1} K^{-1})	Abs Error (κ⊥)
1	1.38e+07	0.11e+07	0.59	0.10
2	1.39e+07	0.11e+07	0.54	0.09
3	1.15e+07	0.09e+07	0.55	0.10
4	1.33e+07	0.11e+07	0.53	0.09
5	1.50e+07	0.11e+07	0.52	0.09
6	1.43e+07	0.10e+07	0.48	0.08
Mean	1.36e+07	0.12e+07 (SD)	0.54	0.04 (SD)

S9 In-plane thermal measurement

To determine the in-plane thermal conductivity of the $Ti_3C_2T_x$ MXene flake, we employed the beam-offset frequency-domain thermoreflectance (FDTR) technique developed previously.[10,11] Owing to the weak thermoreflectance response of the bare $Ti_3C_2T_x$ MXene surface, a ~50 nm-thick Au transducer was selectively deposited on part of the sample surface to ensure a sufficient signal level for reliable phase detection. The deposited Au layer defined a sharp and straight boundary, with the adjacent uncovered region remaining as exposed $Ti_3C_2T_x$. During the beam-offset measurements, the probe beam was focused on the Au transducer near this boundary, whereas the long axis of the line-shaped pump beam was aligned parallel to the Au edge and positioned on the bare $Ti_3C_2T_x$ region. This geometry ensured that the lateral heat flow between the line-shaped pump excitation and the point-like probe detection path occurred predominantly through the uncovered $Ti_3C_2T_x$ region, thereby minimizing parasitic contributions from the Au transducer to the measured in-plane thermal conductance.

The beam-offset FDTR measurements were carried out at six modulation frequencies between 2 and 15.7 kHz. The pump–probe offset was restricted to the range of 8–18 μm, which avoids finite-spot-size effects at small offsets while preserving an adequate signal-to-noise ratio at larger offsets, where the spatial dependence of the measured phase response becomes progressively weaker. At each modulation frequency, the phase lag of the thermally induced oscillation was recorded as a function of pump–probe

offset and fitted using a linear relation. The extracted slopes further present their dependence on the square root of the modulation frequency. A second linear regression was then applied to this frequency-dependent slope, yielding an in-plane thermal diffusivity of $1.4 \times 10^{⁻⁵}$ m^2 s$^{⁻1}$. Combining this diffusivity with the reported volumetric heat capacity of $Ti_3C_2T_x$, $C_v = 3.39 \times 10^{⁶}$ J m$^{⁻3}$ K$^{⁻1}$,[6] gives an in-plane thermal conductivity of 46 ± 6 W m$^{⁻1}$ K$^{⁻1}$.

S10 Qualitative infrared imaging of the $Ti_3C_2T_x$ film

To provide a simple qualitative demonstration of the thermal response of the $Ti_3C_2T_x$ film, infrared imaging was performed using the human hand as a heat source. Figure S8a shows a photograph of the experimental configuration, in which a $Ti_3C_2T_x$ film was attached to the surface of the fist. Figures S8b and S8c show infrared images of the bare fist and the same fist partially covered by the $Ti_3C_2T_x$ film, respectively. A clear apparent thermal contrast is observed in the film covered region in Figure S8c relative to the surrounding skin. However, this contrast should not be interpreted directly as a quantitative temperature reduction or thermal barrier effect. Infrared thermography determines apparent surface temperature from thermal radiation and therefore depends strongly on surface emissivity. Although the same camera emissivity setting of 0.95 was used for the images, the emissivity of the $Ti_3C_2T_x$ film was not independently calibrated under the present conditions. Consequently, the infrared images are presented only as a qualitative proof of concept. Quantitative evaluation of the thermal response would require independent emissivity calibration and controlled radiometric measurements.

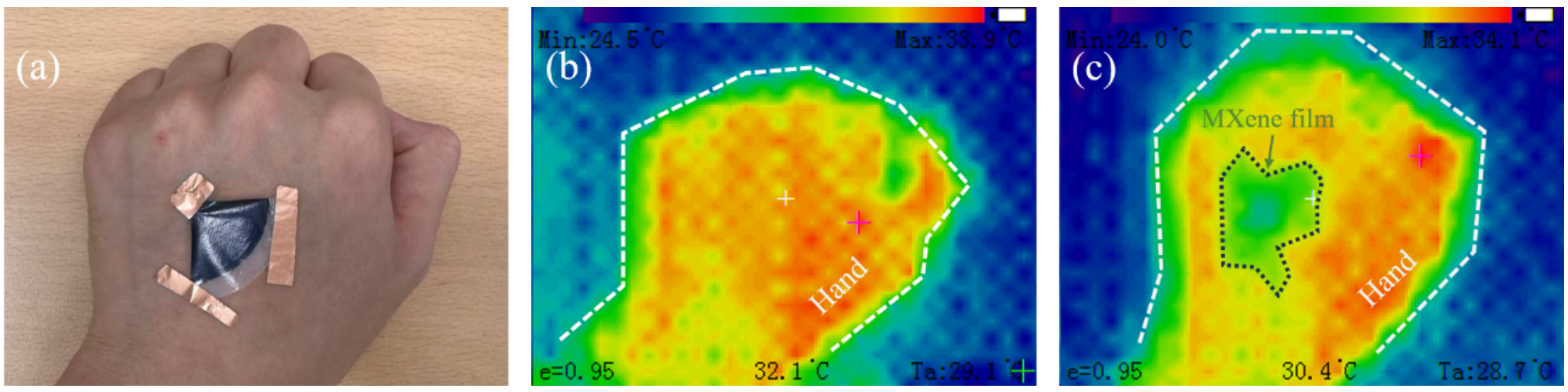

Figure S8 Qualitative infrared demonstration of a $Ti_3C_2T_x$ film on the human hand. (a) Photograph of the experimental configuration, showing the $Ti_3C_2T_x$ film attached to the surface of the fist. (b) Infrared image of the bare fist. (c) Infrared image of the same fist partially covered by the $Ti_3C_2T_x$ film. The film covered region exhibits a clear apparent thermal contrast relative to the surrounding skin. The infrared images were acquired using the same emissivity setting of 0.95. Because the emissivity of the $Ti_3C_2T_x$ film was not independently calibrated, the apparent temperature contrast is presented only as qualitative evidence and should not be interpreted as a quantitative temperature reduction or thermal barrier performance.

Reference

1 A. Schmidt, M. Chiesa, X. Chen and G. Chen, *Rev. Sci. Instrum.*, 2008, **79**, 064902.
2 X. Qian, J. Zhou and G. Chen, *Nat. Mater.*, 2021, **20**, 1188–1202.
3 A. J. Schmidt, X. Chen and G. Chen, *Rev. Sci. Instrum.*, 2008, **79**, 114902.
4 D. G. Cahill, *Rev. Sci. Instrum.*, 2004, **75**, 5119–5122.
5 A. J. Schmidt, R. Cheaito and M. Chiesa, *Rev. Sci. Instrum.*, 2009, **80**, 094901.
6 S. Srivatsa, W. Tokarz, J. Przewoźnik, T. Strączek, K. Grabowski, P. Rutkowski, T. Uhl, J. Kulawik, D. Kata, D. Madej, J. Lis and C. Kapusta, *Materials*, 2024, **17**, 2199.
7 M. Dong, Y. Hu, H. Zhang, E. Bilotti, N. Pugno, D. Dunstan and D. G. Papageorgiou, *Composites Part C: Open Access*, 2024, **13**, 100427.
8 Chas. G. Maier and K. K. Kelley, *J. Am. Chem. Soc.*, 1932, **54**, 3243–3246.
9 C. Kittel and P. McEuen, *Introduction to solid state physics*, John Wiley & Sons, 2018.
10 K. Xu, S. Skorda, P. Xiao, E. Coy, X. Cartoixà, R. Rurali, J. S. Reparaz and A. El Sachat, *Nano Lett.*, 2025, **25**, 16006–16012.
11 K. Xu, J. Guo, G. Raciti, A. R. Goni, M. I. Alonso, X. Borrisé, I. Zardo, M. Campoy-Quiles and J. S. Reparaz, *International Journal of Heat and Mass Transfer*, 2023, **214**, 124376.
